# Streptozotocin Causes Blood–Brain Barrier and Astrocytic Dysfunction In Vitro

**DOI:** 10.3390/cells14211745

**Published:** 2025-11-06

**Authors:** Sarah A. Habib, Mohamed M. Kamal, Mohamed H. Aly, Heba R. Ghaiad, Sherine M. Rizk, William A. Banks, Michelle A. Erickson

**Affiliations:** 1 Biochemistry Department, Faculty of Pharmacy, Cairo University, Kasr El-Aini Street, Cairo 11562, Egypt; sarah.habib@bue.edu.eg (S.A.H.); heba.mosalam@pharma.cu.edu.eg (H.R.G.); sherine.abdelaziz@pharma.cu.edu.eg (S.M.R.); 2 Pharmacology and Biochemistry Department, Faculty of Pharmacy, The British University in Egypt, El Sherouk City, Cairo 11837, Egypt; mohamed.kamal@bue.edu.eg (M.M.K.); mohamed.aly@bue.edu.eg (M.H.A.); 3 Drug Research and Development Group, Health Research Center of Excellence, The British University in Egypt, El Sherouk City, Cairo 11837, Egypt; 4 Biochemistry Department, Faculty of Pharmacy, Ain Shams University, Cairo 11566, Egypt; 5 Institute of Physiology, Christian-Albrechts-University of Kiel, 24118 Kiel, Germany; 6 Department of Medicine, Division of Gerontology and Geriatric Medicine, University of Washington, Harborview Medical Center, 325 9th Avenue, Seattle, WA 98104, USA; wabanks1@uw.edu; 7 Geriatric Research Education and Clinical Center, VA Puget Sound Health Care System, 1660 S. Columbian Way, Seattle, WA 98108, USA

**Keywords:** Streptozotocin, blood–brain barrier, brain endothelial cells, astrocytes, tight junctions, GLUT-1

## Abstract

Streptozotocin (STZ) is an alkylating agent that has neurotoxic effects when injected into the cerebral ventricles (ICV) and also models many other features of Alzheimer’s disease. However, the mechanisms of STZ neurotoxicity are not well understood. In this study, we hypothesized that some of the neurotoxic effects of STZ could be due to direct activities on brain endothelial cells and astrocytes, which are key in forming and supporting the functions of the blood–brain barrier (BBB), respectively. To test this hypothesis, we characterized the changes induced by STZ either in cultures of human-induced pluripotent stem cell (iPSC)-derived brain endothelial-like cells (iBECs), which form an in vitro BBB model, or in primary human astrocytes. We found that STZ at a dosage of 5 mM caused a delayed reduction in the transendothelial electrical resistance (TEER) of iBECs at 7–11 days post-treatment, indicating induction of BBB leakage. Additionally, we observed significant increases in albumin leakage across the monolayer, altered iBEC morphology, and reductions in tight junction proteins, suggesting that STZ causes BBB disruption. We further found that the BBB glucose transporter GLUT-1 was reduced in iBECs, as was the total number of iBECs. In astrocytes, the 5 mM dose of STZ reduced the GFAP signal and total number of cells, suggesting that STZ has anti-proliferative and/or toxic effects on astrocytes. Together, these data support that STZ’s neurotoxic effects could be due, in part, to its direct toxic activities on brain endothelial cells and astrocytes.

## 1. Introduction

Streptozotocin (STZ) is a naturally occurring nitrosourea derived from the *Streptomyces achromogenes* bacterium. It is a DNA-alkylating antineoplastic agent used therapeutically for the treatment of pancreatic islet cell carcinoma [1]. Its widespread use in diabetes research stems from its preferential toxicity toward insulin-producing β-cells after intravenous or intraperitoneal administration [2,3]. This is attributed to its structural similarity to glucose, which allows its intracellular accumulation via the glucose transporter-2 (GLUT-2), but not through other glucose transporters [4]. Once in the cell, STZ exerts its cytotoxic effects through DNA alkylation and oxidative stress induction, ultimately resulting in β-cell death [5].

STZ administered via peripheral routes is thought not to enter the brain because GLUT-2 is essentially absent from brain endothelial cells that form the blood–brain barrier (BBB). However, the diabetic state caused by STZ can result in pathological brain changes such as BBB disruption, neuroinflammation, oxidative stress, and cognitive dysfunction [5,6]. When injected intracerebroventricularly (ICV), STZ does not induce a type 1 diabetic phenotype but models many aspects of Alzheimer’s disease (AD) [7]. AD-related changes in response to ICV-STZ include neuronal loss, impaired brain insulin signaling, neuroinflammation, oxidative stress, increased CNS production of amyloid beta peptide, tau hyperphosphorylation, and cognitive impairment [8,9,10]. Because GLUT-2 expression is mostly absent from the brain, with the exception in a few regions [11], the mechanisms by which STZ induces CNS dysfunction is unclear. Prior studies have shown that STZ is directly toxic to neurons in vitro [12,13], highlighting the utility of in vitro models to delineate mechanisms of ICV-STZ toxicity that contribute to an AD-like brain state.

Dysfunction of the BBB is thought to be a driving pathological change in AD [14] but has been understudied in ICV-STZ models. The vascular BBB is primarily composed of brain endothelial cells and maintained in part by the closely associated endfeet of astrocytes that line most of the brain-facing microvascular surface [15]. The BBB supports physiologic brain functions by restricting the unregulated diffusion of polar substances across vascular membranes and facilitating the selective transport of circulating nutrients, trophic factors, and other substances that support brain functions [16,17]. Brain endothelial cells of the BBB express specialized tight junction proteins (TJPs) such as occludin, claudin-5, and zonula occludens (ZO-1), which seal paracellular spaces between endothelial cells and are crucial for maintaining the barrier’s properties [18]. A highly expressed and essential transporter at the BBB is the glucose transporter protein type-1 (GLUT-1), which is the main insulin-independent transporter of glucose across the BBB [19]. Disrupting the barrier’s integrity leads to increased membrane permeability, resulting in the leakage of potentially harmful substances into the CNS and subsequent inflammatory responses and oxidative stress that contribute to neurotoxicity and neurodegeneration [20]. Additionally, diminished functions of transporters like GLUT-1 at the BBB result in decreased energy supply to the brain, causing metabolic dysfunction and clinical outcomes such as epileptic seizures that can also be associated with AD [18,19]. Evidence supports that GLUT-1 is reduced in brain endothelial cells of humans with Alzheimer’s disease [21].

Although most studies of ICV-STZ’s effects on brain cells have been in vivo, emerging in vitro studies have supported its direct toxicity in neurons [22,23] and shown that STZ can increase activation markers in astrocytes at lower concentrations [24]. However, direct effects of STZ on BBB functions in vitro have not been evaluated. Therefore, we examined STZ for effects on an in vitro model of the human BBB using human-induced pluripotent stem cell (iPSC)-derived brain endothelial-like cells (iBECs) [24,25], as well as its effects on primary human astrocytes. Our primary findings were that STZ induced a delayed and progressive BBB leakage that was associated with reduction in cell numbers, lower levels of TJPs, and loss of GLUT-1 transporter expression. STZ at concentrations required to cause BBB leakage also caused astrocyte loss and morphologic alterations. These findings support that STZ may exert some of its toxic, AD-like effects in the brain by acting directly on brain endothelial cells, causing BBB damage. Additionally, the delayed BBB leakage caused by STZ supports the use of iBECs to study BBB-protective interventions that may be more widely applicable to neurodegenerative conditions involving BBB leakage as a driver.

## 2. Materials and Methods

### 2.1. STZ and Mannitol Preparation

STZ (Cat. No. S0130) and Mannitol (Man) (Cat. No. M4125) were purchased from Sigma-Aldrich (St. Louis, MO, USA). STZ (M.wt = 265.2 g/mole) was used to prepare a stock solution of 0.5 M concentration by dissolving 50 mg of STZ in 377 µL phosphate-buffered saline (PBS) (adjusted pH = 4.5), then dividing into 50 µL aliquots along with 50 µL aliquots of PBS Vehicle (Veh) and storing at −80. For working solutions, a frozen aliquot was thawed, and 1 mM and 5 mM concentrations were prepared in medium immediately before treatment. Each aliquot was only thawed once.

Man (M.wt = 182.172 g/mole) was used to prepare a stock solution of 0.5 M concentration by dissolving 455 mg Man in 5 mL PBS. Afterwards, 1 mM and 5 mM concentrations were used as working solutions.

The concentrations of STZ and Man used were based on works from prior in vivo studies [12,13], and the 5 mM dosage is slightly lower than the estimated distributed concentrations of STZ in rodent CSF post-injection (see Section 4 Discussion). Additionally, the 5 mM concentration of mannitol is much lower than that used to induce BBB opening for CNS drug delivery [26].

### 2.2. Establishment of the In Vitro BBB Model

Human-induced pluripotent stem cells (hiPSCs) from the GM25256 line (Coriell Institute, Camden, NJ, USA) were maintained on Matrigel (Corning, New York, NY, USA, cat no. 356230)-coated plates in E8 Flex medium (ThermoFisher Scientific, Waltham, MA, USA, cat no. A28585-01) to obtain hiPSC-derived brain endothelial-like cells (iBECs) using the Neal et al. protocol [25], and further characterized for the GM25256 line by our lab [27]. hiPSCs were propagated on Matrigel and passaged with Versene (Life Technologies, Carlsbad, CA, USA). As cells neared confluency, they were dissociated into single cells with Accutase (ThermoFisher Scientific, USA cat no. A1110501) and plated on Matrigel at a density of 15,000 cell/well in E8 Flex medium supplemented with 10 µM Rho-associated protein kinase (ROCK) inhibitor Y-27632 (R&D Systems, Minneapolis, ME, USA, cat no. 1254). To initiate differentiation, the medium was changed to E6 (ThermoFisher Scientific, cat no. A1516401) and changed daily for three more days. Afterwards, the medium was changed to human endothelial serum-free medium (HESFM, ThermoFisher Scientific, cat no. 11111044) supplemented with 10 µM retinoic acid (RA; Sigma, St. Louis, MA, USA, cat no. R2625), 20 ng/mL basic fibroblast growth factor (bFGF; Peprotech, Cranbury, NJ, USA, cat no. 100-18B), and 1% B27 supplement (ThermoFisher Scientific, cat no. 17504044). Two days later, differentiated iBECs were Accutase-dissociated and sub-cultured onto 24-well transwell permeable inserts (Corning, cat no. 3470) or tissue culture plates (Corning, cat no. 3513, 3548) coated with 1 mg/mL Collagen IV (Sigma, cat no. C5533) and 5 mM Fibronectin (Sigma, cat no. F1141) in HESFM  +  20 ng/mL bFGF, 10 µM RA, and 1% B27 (day 0). For sub-culturing, iBECs were split at a ratio of 1 well from a 6-well plate to 6 transwells (for TEER and leakage assays), or 6 wells of a 48-well plate (for immunofluorescence), or 2 wells of a 12-well plate (for Western blotting). The medium was changed to HESFM  +  1% B27 after 24 h, and the STZ, Veh, and control treatments occurred three days after the medium change by replacing the medium on both luminal and abluminal sides with the appropriate concentration of STZ, Man, or Veh. The cells then remained in the changed medium for the duration of treatment without further medium changes.

### 2.3. Transendothelial Electrical Resistance (TEER) Measurement

Resistance (Ω) values of iBEC-seeded monolayers on transwells were measured on day 1 post-subculture using an EVOM2 voltammeter (World Precision Instruments, Sarasota, FL, USA) coupled to an ENDOHM cup chamber to confirm the BBB properties of iBECs. Transendothelial electrical resistance (TEER) was calculated via subtracting the resistance (Ω) of the blank transwell, then multiplying by the transwell surface area (0.33 cm^2^). On day 4 post-subculture, TEER was measured again prior to treatment to establish pre-treatment baselines and to arrange the wells into groups such that the starting TEER means were approximately equal and did not significantly differ between groups. The iBECs continued to be tracked daily for 11 days post-treatments; experiments were only conducted on iBECs with pre-treatment TEER values  >  1000 Ω × cm^2^.

### 2.4. iBEC Monolayer Permeability to ^131^I-BSA

Bovine serum albumin (BSA) was labeled with ^131^I using the chloramine-T method and was purified on Sephadex G-10 column. TEER was measured for wells of all groups, then transwells were rearranged so that the average TEER values were almost equal among groups. The media in the luminal and abluminal compartments were replaced by fresh HESFM and 1% B27 media, then incubated for 20 min at 37 °C, which is the time necessary for TEER to stabilize following media change. In order to evaluate the blood-to-brain (luminal-to-abluminal) transport, 100 µL of the luminal (upper) compartment was replaced with 100 µL of input medium containing 3(10^6^) counts per minute (cpm) of ^131^I-BSA. At specific incubation times (20, 60, 90 min), 500 µL of medium from the abluminal (lower) compartment was collected into tubes and then replaced by fresh HESFM  +  1% B27 medium. The collected fractions were acid-precipitated in 15% trichloroacetic acid (TCA) and centrifuged at 4200× *g* for 10 min. The supernatant was discarded, and the pellets were counted on a Wizard 2 gamma counter (Perkin Elmer) to reflect the intact ^131^I-BSA that crossed the in vitro BBB.

The method of Dehouck et al. [28] was used to calculate the permeability–surface area coefficient (PS) of the endothelial monolayer (Pe) in which the clearance was described as µL of radioactive substance transported from the donor to the receptor chamber. This was calculated by comparing the final level of radioactivity in the abluminal compartment with the initial level of radioactivity of the injectate added to the luminal compartment.

Clearance (μL) = [C]c × Vc/[C]_L_ (1)

where [C]_C_ is the concentration of radioactivity in the abluminal compartment (in units of CPM/µL), Vc is the volume of the abluminal compartment in µL, and [C]_L_ is the initial concentration of radioactivity in the luminal compartment (in units of CPM/µL). A graph was plotted for the cleared volume against time, and linear regression was used to estimate the slope.

The PS value for the iBECs monolayer (PSe) was calculated as follows:

1/PSapp  =  1/PSmembrane  +  1/PSe(2)

where PS is the permeability  ×  surface area product (in µL/min), PSapp is the slope of the clearance curve for the iBEC monolayer plus the Transwell^®^ membrane, and PS_membrane_ is the slope of the clearance curve for the Transwell^®^ membrane without iBECs.

PS_e_ values were divided by the surface area of the Transwell^®^ inserts (0.33 cm^2^) to generate the endothelial permeability coefficient (Pe) in (µL/min/cm^2^).

### 2.5. Astrocyte Preparation

Poly-l-lysine (PLL) (1 mg/mL) (ScienCell cat no. 0403, Carlsbad, CA, USA) was prepared by adding 15 µL to 10 mL H_2_O, then filter-sterilized to coat the culture vessel and incubated at 37˚C overnight (or at least 1 h). Astrocyte medium (complete medium) was prepared using astrocyte medium (ScienCell, cat no. 1801, LOT 35005, USA), 2% fetal bovine serum (FBS; ThermoFisher, cat no. A5256701, USA), and 1% B27, freshly added prior to use. A vial of human astrocytes (ScienCell, cat no. 1800, USA) at passage 3 containing 2 × 10^6^ cells was thawed and added dropwise to 10 mL of complete medium in the culture vessel. Cells were fed the following day with the same media. Medium was changed every three days until reaching 70% confluency, then changed every other day until cells reached 90% confluency. For sub-culturing, cells were washed with PBS, trypsinized using 0.25% Trypsin–EDTA (ScienCell, cat no. 0113, USA) for 5 min at 37 °C, washed twice with 1 mL trypsin/EDTA neutralization solution (TNS), then collected in 1 mL FBS. Cells were spun at 200 g for 5 min at 25 °C, then seeded into 48-well plates at a concentration of 40,000 cells/mL and maintained until the following day in the appropriate media. The plated cells were treated with either complete medium only or complete medium with 1 mM STZ or 5 mM STZ and incubated for five days without further medium changes. The 5-day incubation period was determined by daily phase-contrast microscopy to monitor astrocyte morphology and cell numbers post-treatment, with five days determined as optimal for visualizing initial STZ-induced alterations.

### 2.6. Immunofluorescence Analyses

For immunofluorescent analysis, iBECs or astrocytes were seeded on 48-well plates and incubated with STZ, Man, or Veh. The iBECs were treated for 11 days, and astrocytes were treated for 5 days. The 48-well plated iBECs and astrocytes were washed once with PBS (ThermoFisher Scientific, cat no. 70011044, USA) and then fixed in a 1:1 methanol/acetone mixture at 4 °C for 15 min. Fixation occurred after 11 days of STZ treatment for iBECs and after 5 days of STZ treatment for astrocytes. Afterwards, wells were washed three times with PBS for 5 min each. Then, 5% normal donkey serum (Jackson ImmumoResearch, West Grove, PA, USA, cat no. 017-000-121) in 0.1% Triton-X100 (T-X100) (Sigma, cat no. ×100, USA) dissolved in PBS was added at room temperature (RT) for 1 h to allow blocking. Wells were then washed three times with PBS (1 mL/well) for 5 min each. Then, iBECs were incubated with the following primary antibodies: glucose transporter-1 (GLUT-1) 1:50 (Millipore, cat no. 07-1401, Burlington, MA, USA), claudin-5 (ThermoFisher, cat no. 35-2500, USA) 1:50, and zonula occludens-1 (ZO-1) (ThermoFisher, cat no. 61-7300, USA) 1:25, and astrocytes were incubated with primary antibody glial fibrillary acidic protein (GFAP) 1:1000 (Millipore, cat no. AB5541, USA) in phosphate-buffered normal antibody diluent (NAD) (ScyTek, cat no. ABB500, Logan, UT, USA) at 4 °C overnight.

The wells were then washed three times with PBS  for 5 min each and incubated with secondary anti-mouse Alexa Fluor^®^ 594 (Jackson ImmunoResearch, cat no. 715-585-150, West Grove, PA, USA) for GLUT-1, claudin-5, and GFAP with dilution 1:200, and secondary anti-rabbit Alexa Fluor^®^ 488 (Jackson ImmunoResearch, cat no. 711-545-152, USA) for ZO-1 with dilution 1:200, as well as DAPI (ThermoFisher Scientific, cat no. 62248, USA) 1:5000 in NAD at RT for 1 h. Finally, wells were washed three times for 5 min each and placed in PBS to be imaged using an Axiovert 7 Inverted Microscope (Zeiss, Oberkochen, Germany). For each group, three independent images were taken per well. Zen 2.3 image analysis software was used to quantify fluorescence area values.

### 2.7. Protein Extractions and Immunoblots

iBECs plated on 12-well plates and astrocytes plated on 6-well plates were treated with STZ, Man, or Veh for 11 days (iBECs) or 5 days (astrocytes). After treatment, cells were washed with PBS, then scraped in RIPA buffer (deionized water, 1.5 M NaCl, 1 M Tris-HCl, 5 mM MgSO4) supplemented with one tablet protease inhibitor/10 mL buffer (Sigma, cat no. P8340, USA) and 1% phosphatase inhibitor (Sigma, cat no. P5726, USA). Cell lysates were kept at −80 °C. Samples were thawed on ice and centrifuged at 20,000 rcf at 4 °C for 5 min. Bradford assay 1/100 (ThermoFisher Scientific, cat no. 23200, USA) was used to measure the protein concentrations of cell lysates via standardization against a protein standard curve from known concentrations of bovine serum albumin. To prepare the samples for electrophoresis, 4 × LDS buffer (Novex, cat no. NP007), 10 × dithiothreitol (reducing agent) (Novex, cat no. NP0009, Barberton, OH, USA), and distilled water were added to the samples, which were then put in a heat block for 10 min at 70 °C to allow denaturation of samples. iBEC or astrocyte samples containing 30 μg of protein were injected into Express Plus PAGE precast gels (GeneScript, cat no. M41210, Piscataway, NJ, USA). The samples were allowed to run at 100 volts for 30 min, then 150 volts for another 30 min. The gels were washed with water, then the iBlot Dry Blotting System (Invitrogen, Waltham, MA, USA) was used to transfer the samples onto nitrocellulose membranes (Invitrogen, cat no. IB301002, Waltham, MA, USA). Afterwards, the nitrocellulose membranes were soaked for 45 min in Tris-buffered saline with 0.1% Tween-20 (TBS-T) supplemented with 5% BSA (Sigma, cat no. A7030) on a rocker at RT to allow membrane blocking. Membranes were incubated overnight in primary antibody solutions (see Table 1) prepared in 5% BSA/TBS-T at 4 °C. Membranes were then washed three times with TBS-T for 5 min each, followed by incubation with secondary antibodies at RT for 30 min in Table 1. Membranes were then washed three times with TBS-T for 5 min each, followed by single 1 min TBS wash. For the bands to be developed, West Pico chemiluminescence reagent (ThermoFisher Scientific, cat no. PI-34078, USA) was applied and the bands were visualized with the ImageQuant LAS4000 (Cytiva, Marlborough, MA, USA) or the Amersham™ ImageQuant 800 (Cytiva, USA). Quantification of the band volumes was performed with ImageQuant 8.2 TL 1D Gel Analysis Software (Cytiva, USA).

### 2.8. Statistical Analyses

All the data were subjected to Shapiro–Wilk normality tests. Parametric data are presented as mean ± standard error of mean (SEM). Comparisons between means were performed by either *t*-test (for two samples) or one-way analysis of variance (ANOVA) test for more than two samples, followed by Tukey’s post hoc test, or two-way ANOVA followed by Tukey’s post hoc test. Significant differences were applied at *p*-values < 0.05. All statistical analyses and figures were carried out using Graph Pad Prism-version 9, USA.

## 3. Results

### 3.1. Effects of STZ on the In Vitro iBEC BBB Model: TEER Measurements

**Figure 1:**
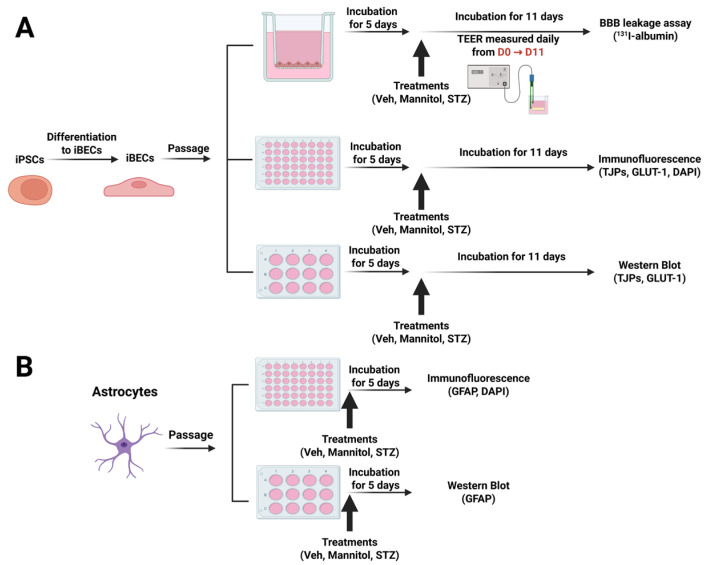
(**A**) Experimental designs for treatments of human iBECs and (**B**) primary human astrocytes and endpoint measurements. Figure created with BioRender.

**Figure 2:**
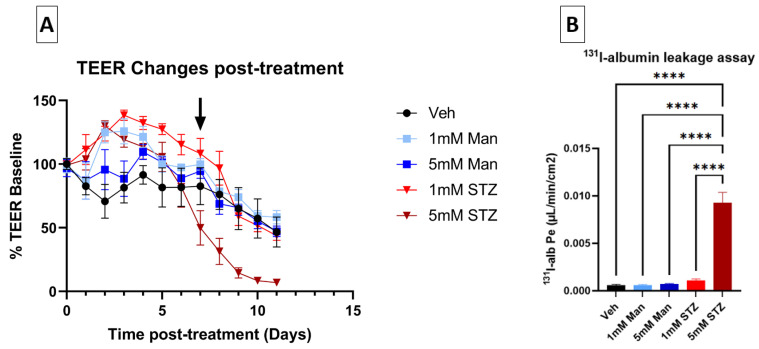
Effects of STZ on leakage of the iBEC monolayer. (**A**) Effect of STZ on TEER levels of iBECs. TEER values were calculated as a percentage of pre-treatment baseline expressed as mean ± SEM and compared between the five groups as follows: Vehicle (Veh), 1 mM Mannitol (Man), 5 mM Man, 1 mM STZ, and 5 mM STZ for 11 days. The average pre-treatment baseline TEER for all groups was 2833.7 ± 436.7 Ω × cm^2^. D7 was the start of the significant decline in the 5 mM STZ group compared with the 5 mM Man group (black arrow), and D8 was the start of the significant decline in the 5 mM STZ group compared with the Veh group. Statistics are shown in Table 2 (*n* = 7–11 transwells per group). (**B**) ^131^I-albumin leakage assay through iBECs. Luminal-to-abluminal transport was assessed by measuring the leakage of ^131^I Alb across the monolayer on D11 after subculture. One differentiation was performed with *n*  =  7 transwells per group. The 5 mM STZ group showed a significantly higher (Pe) when compared with all other groups with a **** *p*  <  0.0001, as determined by Tukey’s multiple comparisons test.

STZ has previously been shown to cause neuronal toxicity at 2–5 mM concentrations [12], comparable to STZ concentrations achieved in the brain following ICV injections [29,30]. We hypothesized that STZ would also cause BBB dysfunction at similar concentrations of STZ in vitro [13]. To test this hypothesis, iBECs grown on Transwells^®^ were used as an in vitro BBB model (see Section 2 Methods) and treated on both the luminal and abluminal sides with 1 mM or 5 mM of either STZ, Man, or Veh (Figure 1A). Man served as a control for osmotic stress, which could be a non-specific effect of STZ treatment. The TEER, a measure of BBB paracellular integrity, was measured before treatment and tracked daily for 11 days to follow the development and persistence of BBB dysfunction. As shown in Figure 2A and Table 2, TEER values (% of pre-treatment baseline) of the 5 mM STZ group decreased significantly vs. the Vehicle (Veh) group from day (D)8 to D10 (Veh D8, 76.2 ± 11.8; 5 mM STZ D8, 31.5 ± 10.3; Veh D9, 65.1 ± 16.6; 5 mM STZ D9, 14.5 ± 4.1; Veh D10, 57.4 ± 15.4, 5 mM STZ D10, 8.4 ± 1.6, *p* < 0.05). Moreover, the 5 mM STZ group was significantly lower than 5 mM Mannitol starting from D7 to D10, (5 mM STZ D7, 49.9 ± 13.5; 5 mM Mannitol D7, 94.6 ± 5.8; 5 mM STZ D8, 31.5 ± 10.3; 5 mM Mannitol D8, 68.8 ± 8.3; 5 mM STZ D9, 14.5 ± 4.1; 5 mM Mannitol D9, 65.8 ± 6; 5 mM STZ D10, 8.4 ± 1.6; 5 mM Mannitol D10, 55.2 ± 5.9, *p* < 0.05). The 5 mM Mannitol group did not show any significant difference when compared with the Veh group. These data indicate that STZ is causing BBB leakage by a mechanism other than that of generalized osmotic stress.

### 3.2. Effects of STZ on the In Vitro iBEC BBB Model: Transport of ^131^I Alb

To confirm whether STZ causes BBB leakage to a larger (65 kDa) molecular weight tracer, we quantified transendothelial transport of ^131^I-alb across iBECs monolayers on D11 post-treatment (Figure 1A). The permeability–surface area coefficient (Pe) for luminal-to-abluminal transport was measured. Corroborating the TEER data, Figure 2B shows that 5 mM STZ induced a significantly higher Pe of albumin when compared with all other groups (*p* <  0.0001), whereas the 1 mM STZ group had no statistically significant effect on albumin leakage across the iBEC monolayer.

### 3.3. Effects of STZ on the iBEC BBB Model: iBEC Numbers, Tight Junctions, and GLUT1

**Figure 3:**
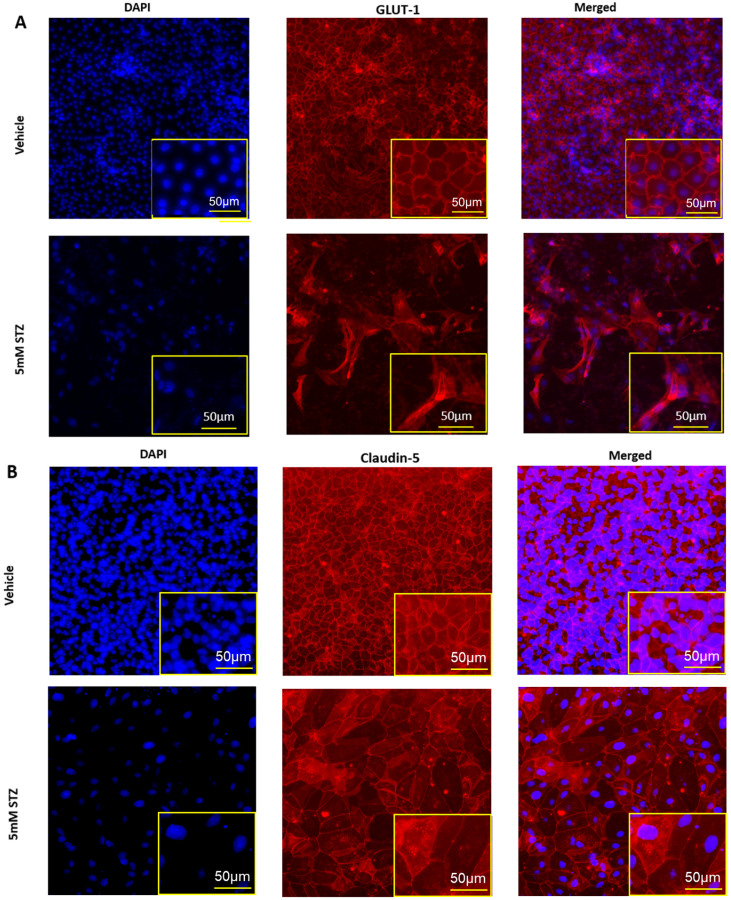

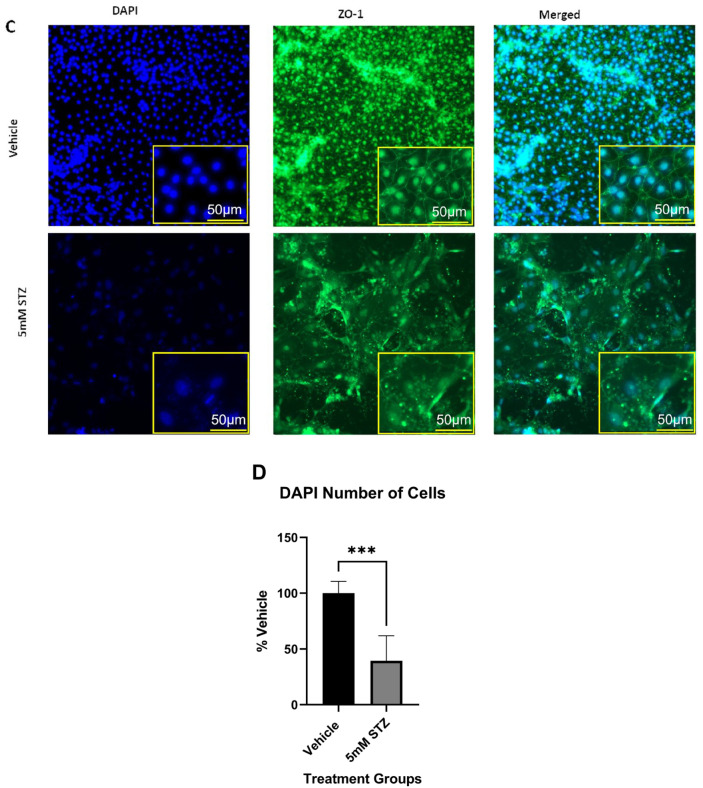
Immunocytochemistry of TJPs in GM25256 iBECs and immunofluorescent analysis of DAPI-stained nuclei. (**A**–**C**) Representative immunocytochemistry (middle column) of GLUT-1 (red), claudin-5 (red), and ZO-1 (green) in Vehicle (the upper rows) and 5 mM STZ (the lower rows) treated cells after 11 days of subculture. Cells were counterstained with DAPI (the left column) (blue), and the overlay is shown (right column). (**D**) Immunofluorescent analysis of DAPI-stained nuclei among the Vehicle and 5 mM STZ groups. Each data point represents the mean ± SEM of three random fields of view per well, *n* = 6 wells/group, *** *p*  <  0.001 as determined by a two-tailed Student’s *t*-test.

To further characterize potential mechanisms of BBB dysfunction that are relevant to neurodegenerative diseases like AD, we evaluated how STZ treatment affected the total numbers of iBECs, expression levels and localization of the TJPs claudin-5 and ZO-1, as well as the BBB glucose transporter GLUT-1 11 days post-treatment. In Figure 3A–C, we show that STZ causes a significant decline in the number of DAPI-stained nuclei, which is quantified in Figure 3D. Whereas junctional localization was observed for GLUT-1, claudin-5, and ZO-1 for Veh-treated cells, STZ induced a mislocalization of all three proteins, as well as some gaps in protein signal. Additionally, cells took on an enlarged morphology compared with the Veh group.

**Figure 4:**
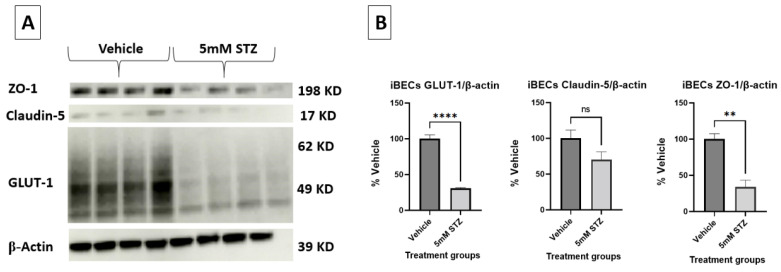
(**A**,**B**) Representative immunoblots and immunoblot quantification of claudin-5, ZO-1, and GLUT-1 expression in the Vehicle and the 5 mM STZ groups. Expression was calculated relative to β-actin. Representative immunoblots (**A**) and quantified bands (**B**). Mean ± SEM of the quantified band intensities of (**A**) were normalized to the β-actin loading control and data were presented as a % of vehicle treatment. *n* =  4 wells for each group analyzed by two-tailed *t*-test. GLUT-1 in the 5 mM STZ group was significantly lower than the Vehicle group with **** *p*  <  0.0001, as well as ZO-1 showing ** *p*  <  0.01, while claudin-5 was not significantly (ns) lower than the Vehicle group.

To further corroborate the effects of STZ on TJPs and GLUT-1, we assessed the semi-quantitative changes in these proteins via immunoblot analysis (Figure 4A,B). Quantification of the expressed bands was calculated relative to β-actin. For GLUT-1, the expression of the 5 mM STZ group (31 ± 0.8) was significantly lower than the Veh group (100 ± 5.6) at *p*  <  0.0001. For ZO-1, the 5 mM STZ group (34 ± 9.3) was also significantly lower than the Veh group (100 ± 7.5) at *p*-value  <  0.001, whereas claudin-5 showed a decline in the 5 mM STZ group (70.3 ± 10.9) compared with the Veh group (100 ± 11.6), but this was not statistically significant.

### 3.4. In Vitro Effects of STZ on Cultured Astrocytes

**Figure 5:**
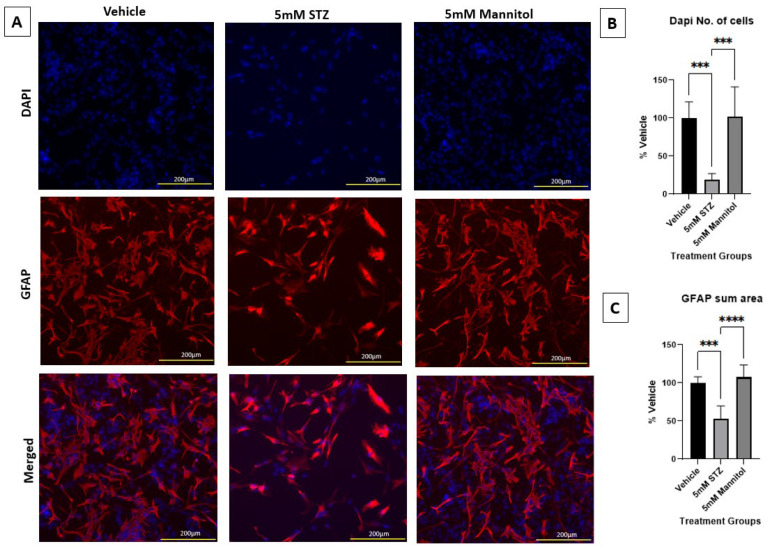
Immunocytochemical staining of astrocytes and immunofluorescent analyses. (**A**) Representative immunocytochemistry of GFAP (red) and DAPI (blue) for Vehicle (left column), 5 mM STZ (middle column), and 5 mM Mannitol (right column) groups after five days of subculture. Nuclei were counterstained with DAPI (blue, uppermost row), GFAP (red, middle row), and the merged images are shown in the lowermost row. (**B**) Mean fluorescence intensities of DAPI among the Vehicle, 5 mM STZ, and 5 mM Mannitol groups. Results show that the 5 mM STZ group was significantly lower than the Vehicle group *** *p*  <  0.001, and the 5 mM Mannitol group was significantly higher than the 5 mM STZ group *** *p*  <  0.001. The 5 mM Mannitol group did not show any significant difference compared with the Vehicle group. Each data point represents the average of three random fields of view per well, *n* = 6 wells/group, and the data are presented as the mean ± SEM, normalized as a % of vehicle. Data were analyzed by one-way ANOVA followed by Tukey’s multiple comparisons test. (**C**) Total GFAP area of the Vehicle, 5 mM STZ, and 5 mM Mannitol groups (mean ± SEM normalized as a % of vehicle). Results show that the 5 mM STZ group was significantly lower than the Vehicle group, *** *p*  <  0.001, and the 5 mM Mannitol group was significantly higher than the 5 mM STZ group, **** *p*  <  0.0001. The 5 mM Mannitol group did not show any significant difference compared with the Vehicle group. Each data point represents the average of three random fields of view per well, *n* = 6 wells/group. Data were analyzed by one-way ANOVA followed by Tukey’s multiple comparisons test.

Astrocytes are an important component of the BBB and support its functions. ICV treatment of STZ in vivo has been shown to increase astrocyte reactivity and lower astrocyte arborization [12,29,30]. To investigate the direct effects of STZ, we treated primary human astrocytes with 5 mM STZ or Man as an osmotic control, based on the dosage found to induce BBB leakage (Figure 2). Figure 5A shows that astrocytes treated with 5 mM STZ had significantly fewer DAPI-stained nuclei and a smaller GFAP area compared to both the Veh and the 5 mM Man groups (Figure 5B,C). As longer incubations with STZ resulted in a nearly complete loss of astrocytes, we did not evaluate astrocytes at later time points.

**Figure 6:**
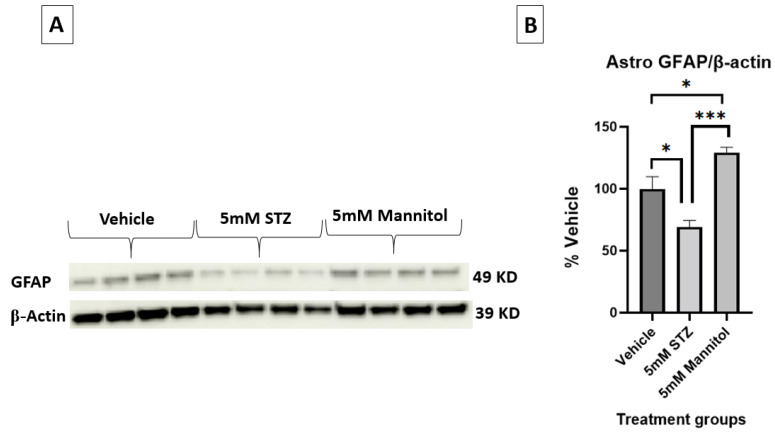
(**A**,**B**) Representative immunoblots and quantification of GFAP in astrocytes treated with Vehicle, 5 mM STZ, or 5 mM Mannitol. *n* = 4 wells/group; bands were normalized to β-actin and presented as % of vehicle. Data are shown are the mean ± SEM analyzed by one-way ANOVA and Tukey’s multiple comparison test, * *p* < 0.05, *** *p* < 0.001.

Additionally, Figure 6A,B show the immunoblots of astrocytic GFAP expression, validating the significantly lower expression in the 5 mM STZ group (69.2 ± 5.3) compared with both the 5 mM Man (129.3 ± 4.2) and Veh groups (100 ± 9.8) with * *p*  <  0.01, *** *p*  <  0.001, respectively.

## 4. Discussion

In this study, we aimed to test our hypothesis that STZ can disrupt the BBB by characterizing the in vitro effects of STZ on iBECs and astrocytes. Our main findings were that STZ at the higher 5 mM concentration induced a latent reduction in BBB integrity as measured by TEER and albumin leakage. Along with this loss of integrity, we found that there were reduced numbers of iBECs, enlarged cell morphology, and loss of TJPs and GLUT-1 in the iBECs in response to STZ treatment. We also saw reduced numbers of astrocytes in response to STZ. Importantly, 5 mM Man did not cause the STZ phenotype, indicating that STZ’s effects are not simply due to osmotic stress on the cells. Our interpretation of these phenotypes, limitations of the study, and future directions are discussed below.

Cell loss and altered morphology were a common phenotype of iBECs and astrocytes with the 5 mM dose of STZ treatment. This dosage is similar to the range found to be toxic to primary rodent neurons in vitro [13]. In vivo ICV-STZ models commonly use a dosage of STZ at a concentration of 3 mg/kg, given to mice in a volume of 3 µL [31,32]. Taking the average weight of a mouse to be about 25 g, this amounts to a 94 mM concentration of STZ in the injectate. Presuming that STZ rapidly distributes within the CSF, and that mouse CSF volume is approximately 37 µL, the average distributed concentration would be about 7 mM prior to clearance. Therefore, the dosage we used in vitro is slightly lower than that used in vivo (ICV) to model the neurodegenerative aspects of AD. Cell death from STZ is thought to first require cellular uptake by GLUT-2, which is the mechanism by which peripherally administered STZ selectively kills cells of the pancreas, causing a type 1 diabetes phenotype [1,2,3]. Once in the cells, STZ causes DNA damage, which engages poly ADP-ribose synthetase and causes further metabolic dysfunction through the depletion of nicotinamide adenine dinucleotide, an essential cofactor in ATP production [33]. In the brain, GLUT-2 expression is largely restricted to specific populations of astrocytes and tanycytes of the hypothalamus [34]. In primary human astrocytes, GLUT-2 expression levels have been detected, albeit at very low levels relative to GLUT-1 and GLUT-3 [35]. However, GLUT-2 was not detected in a previous transcriptomic analysis of iBECs that were differentiated and cultured using the same methods as described herein [36]. Therefore, the mechanisms of cellular uptake of STZ may involve GLUT-2 for astrocytes but are unclear for iBECs. GLUT-2 independent uptake mechanisms should be explored as a future direction in both cell types.

In astrocytes, we observed that STZ caused a reduction in cell numbers as indicated by the reduction in DAPI-positive nuclei, which could be due to cell death and/or an anti-proliferative effect. Levels of GFAP, a marker of astrocyte reactivity, were also reduced with STZ. Interestingly, mannitol caused a significant increase in GFAP, which could indicate increased reactivity in the presence of osmotic stress. In vivo, ICV-STZ was reported to cause alterations in astrocyte morphology and upregulation of astrocyte reactivity markers such as GFAP [29]. However, a more comprehensive regional analysis of GFAP levels in brains found that GFAP increases occurred in some brain regions (cerebral cortex, basal ganglia, and hippocampus), whereas others decreased (entorhinal cortex) or were unchanged (hypothalamus) [37]. This potential disparity of in vitro vs. in vivo findings may be due to much higher numbers of GFAP+ astrocytes at baseline in vitro vs. in vivo, greater vulnerability of cultured astrocytes in a more reactive state to STZ, astrocyte responses to STZ being influenced by the native brain environment and integrated responses of other cell types, or other inherent properties of the astrocytes. Presently, we are not aware of in vivo studies that evaluated whether there was a total astrocyte loss with STZ. Such analysis would likely require the use of pan-astrocyte markers like ALDH1L1 or NDRG2 because, in vivo, not all astrocytes are GFAP+. In AD, it has been purported that a subset of astrocytes do undergo cell death processes including apoptosis or ferroptosis, which are largely driven by DNA damage and oxidative stress, respectively [38]. DNA damage and oxidative stress are also major mechanisms driving STZ toxicity [39]. Because astrocytes are key supporters of neuronal and BBB function, their loss or dysfunction can contribute to aspects of AD [34,37].

As iBECs are seeded at confluence and generally become quiescent over time in culture [27], we interpret the reduction in DAPI+ cells in the iBEC model to be due to cell death. However, the mean TEER values of the STZ group at the endpoint exceeded 200 Ω x cm^2^, which is a TEER value similar to that ideally achieved in primary in vitro BBB models, indicating that the monolayer remained intact. TEER would be expected to be near-zero if cell death were causing gaps in the monolayer. The apparent enlargement of iBECs may be compensatory to repair barrier function by restoring cell–cell contacts. BBB leakage can occur by different modes, such as paracellular, transcellular/vesicular, and degenerative mechanisms, which involve distinct molecular processes [40]. Claudin-5 and ZO-1 are among the major tight junction proteins of the BBB, controlling the paracellular permeability of the BBB [41]. The reduction and mislocalization of the TJPs claudin-5 and ZO-1 that we observed support that one mode of BBB leakage caused by STZ is paracellular, via loss of functional TJPs. Whereas total protein levels of ZO-1 (measured by Western blot) significantly decreased with the 5 mM STZ treatment, the reduction in claudin-5 was less robust and not statistically significant. The staining data corroborate that ZO-1 appeared to be more severely mislocalized than claudin-5, which may indicate greater sensitivity of ZO-1 to STZ-induced damage. Degenerative modes of BBB leakage were also apparent, based on the microscopic findings of cell loss and cellular enlargement, and the large increases in albumin leakage which could occur either via larger paracellular leaks, or increased levels of pinocytosis, that are typically suppressed at the BBB. Another important finding in the iBEC model is that TEER was not significantly reduced by STZ until day 7 post-treatment. As high TEER requires a confluent monolayer and robust, highly functional TJPs, we interpret this to mean that TJP dysfunction and/or cell loss did not occur sooner than day 7 post-treatment. In vivo, peripheral treatments of STZ can cause pancreatic cell death within 24–72 h [42], and the effects of ICV-administered STZ is reported to occur in phases [9]. For example, decreases in reference memory are noted as soon as 3 h post-ICV injection, and neuronal death as measured by Fluorojade C is detectable within 1 day; however, memory impairments worsen over time and it takes weeks to months for alterations in amyloid beta and p-tau levels to appear [36,43]. Therefore, future in vivo studies should evaluate the latency and persistence of BBB dysfunction, which could inform the timing of processes that drive the pathology and ideal windows for interventions. Another notable finding is that the TEER increased in response to STZ at earlier time points (2–5 days) post-treatment. We posit that this reflects initial compensatory responses of the iBECs to the STZ stressor, which are eventually overcome in the 5 mM treatment group. Additionally, the latency in this in vitro model supports its potential for studying therapeutic interventions following a toxic insult. Currently, brain endothelial cell loss has not been investigated using in vivo STZ models, but is an important future direction given our in vitro findings, particularly because brain endothelial damage and apoptosis have been reported in AD [9,42].

A prevailing hypothesis is that a driver of neurotoxicity in the ICV-STZ model is glucose hypometabolism, which is also observed in AD [9]. The brain’s uptake of glucose is mainly insulin-independent and largely driven by the GLUT-1 transporter in brain endothelial cells and astrocytes, and the GLUT-3 transporter in neurons [35,43]. Although neuronal hypometabolism and neurodegeneration are thought to be major contributors to reductions in brain glucose uptake observed in AD [14], GLUT-1 dysfunction at the BBB could also account for this [44]. Protein levels of brain microvascular GLUT-1 are shown to be reduced in AD [21,45,46]. In animal models, reductions in brain endothelial GLUT-1 cause BBB leakage and microvascular degeneration [47]. GLUT-1 levels at the BBB specifically have not yet been evaluated in ICV-STZ models. In vitro; however, we have shown that STZ induces reduced protein levels and mislocalization of GLUT-1 in iBECs. Thus, the reduction in GLUT-1 could be an additional AD-relevant mechanism by which STZ could cause BBB dysfunction.

In summary, our results provide the first in vitro evidence that STZ can cause multiple modes of BBB dysfunction that include the loss and mislocalization of TJPs, loss and mislocalization of GLUT-1, cellular degeneration that is associated with BBB leakage, and astrocyte loss/dysfunction. A limitation of our study is that our time-dependent characterization of BBB phenotypic changes was limited to measures of TEER, whereas we do not know if the other changes occur gradually/concurrently, or in sequence. We also do not yet know the primary mechanisms by which STZ reduces cell numbers (e.g., apoptosis, ferroptosis, necrosis, etc.) in iBECs or astrocytes. However, our initial findings provide the basis for the design of future studies that could further characterize aspects of STZ-mediated BBB dysfunction in vitro and in vivo, as well as interventions to prevent such BBB dysfunctions.

## 5. Conclusions

The complexity and limitations of in vivo animal models necessitate developing predictive in vitro models of human BBB dysfunction, which serve as a critical bridge between preclinical studies and clinical trials and provide novel insights into disease mechanisms that could advance drug development. In this study, a new in vitro model for BBB dysfunction was established using STZ. The delayed progression of BBB leakage following STZ treatment offers a therapeutic window that is ideal for testing novel treatments for neurodegenerative diseases related to BBB disruption, such as AD.

## Figures and Tables

**Table 1:** Primary and secondary antibodies of the immunoblots.

**iBECs**	**1° AB**	**1° AB** **Conc.**	**Vendor and Cat. No.**	**2° AB**	**2° AB** **Conc.**
	GLUT-1	1:10,000	(Millipore, cat no. 07-1401, Burlington, MA, USA) LOT: 3845652	Rabbit	1:5000
	Claudin-5	1:1000	(Invitrogen, cat no. 35-2500, USA) LOT: WC321145	Mouse	1:5000
	ZO-1	1:1000	(Proteintech, cat no. 21773-1-AP, Rosemont, IL, USA) LOT:00106231	Rabbit	1:5000
**Astrocytes**	**1° AB**	**1° AB** **Conc.**	**Vendor and Cat. No.**	**2° AB**	**2° AB** **Conc.**
	GFAP	1:2000	(Cell Signaling Technology, cat no. 3670S, Danvers, MA, USA) ref:GA5 lot.6	Mouse	1:5000
	β-Actin	1:5000	(Abcam, cat no. ab49900, Cambridge, UK) 1043090-2	Mouse	1:5000

**Table 2:** Adjusted *p*-values from the statistical analysis of Figure 1A by two-way ANOVA and Tukey’s multiple comparisons test. Lightly shaded cells indicate a significant increase (green) or decrease (red) with treatment compared to the group on the left (Vehicle (Veh), Mannitol (Man), or 1 mM STZ). Darkly shaded cells indicate a trend (0.05 < *p* < 0.1) toward an increase (green) or decrease (red) compared to the group on the left.

Comparison	Adjusted *p*-Value											
	Day 0	Day 1	Day 2	Day 3	Day 4	Day 5	Day 6	Day 7	Day 8	Day 9	Day 10	Day 11
Veh vs. 1 mM Man	>0.9999	0.9987	0.0015	0.0172	0.2238	0.695	0.8063	0.7481	0.9998	0.9792	>0.9999	0.9474
Veh vs. 5 mM Man	0.9995	0.9977	0.41	0.9882	0.7149	0.6342	0.9874	0.919	0.985	>0.9999	>0.9999	>0.9999
Veh vs. 1 mM STZ	>0.9999	0.1499	0.0003	<0.0001	0.0111	0.0027	0.0611	0.2463	0.4714	0.9953	0.9966	0.9998
Veh vs. 5 mM STZ	>0.9999	0.4423	<0.0001	0.0236	0.4052	0.3126	>0.9999	0.0711	0.0037	0.0122	0.017	0.0869
1 mM Man vs. 1 mM STZ	>0.9999	0.4083	>0.9999	0.9005	0.941	0.3076	0.723	0.9754	0.7004	0.8746	0.9892	0.8908
5 mM Man vs. 5 mM STZ	0.9998	0.769	0.1125	0.1982	0.9986	0.9983	0.9891	0.0157	0.0689	0.0106	0.0259	0.0797
1 mM STZ vs. 5 mM STZ	>0.9999	0.9748	0.9883	0.5451	0.568	0.4141	0.0646	<0.0001	<0.0001	0.0391	0.048	0.132

## Data Availability

The raw data supporting the conclusions of this article will be presented by the authors upon request.

## Abbreviations

The following abbreviations are used in this manuscript:

STZ	Streptozotocin
Man	Mannitol
Veh	Vehicle
AD	Alzheimer’s disease
BBB	Blood–brain barrier
hiPSC	Human-induced pluripotent stem cells
iBECs	Induced brain endothelial cells
TEER	Transendothelial electrical resistance
CPM	Counts per minute
T-X100	Triton-X100
GLUT-1	Glucose transporter-1
GLUT-2	Glucose transporter-2
ICV	Intracerebroventricularly
TJPs	Tight junction proteins
ZO-1	Zonula occludens
PBS	Phosphate-buffered saline
bFGF	Basic fibroblast growth factor
(ROCK) inhibitor	Rho-associated protein kinase (ROCK) inhibitor
HESfM	Human endothelial serum-free medium
BSA	Bovine serum albumin
TCA	Trichloroacetic acid
Pe	Permeability coefficient
PLL	Poly-l-lysine
FBS	Fetal bovine serum
TNS	Trypsin/EDTA neutralization solution
RT	Room temperature
GFAP	Glial fibrillary acidic protein
NAD	Normal antibody diluent
MFI	Mean fluorescence intensity
SEM	Standard error of mean
ANOVA	Analysis of variance
